# Crosslinked Hyaluronan Electrospun Nanofibers for Ferulic Acid Ocular Delivery

**DOI:** 10.3390/pharmaceutics12030274

**Published:** 2020-03-17

**Authors:** Maria Aurora Grimaudo, Angel Concheiro, Carmen Alvarez-Lorenzo

**Affiliations:** Departamento de Farmacología, Farmacia y Tecnología Farmacéutica, I+D Farma (GI-1645), Facultad de Farmacia and Health Research Institute of Santiago de Compostela (IDIS), Universidade de Santiago de Compostela, 15782-Santiago de Compostela, Spain; angel.concheiro@usc.es

**Keywords:** ocular inserts, electrospinning, hyaluronan, ferulic acid, ε-polylysine, nanofibers

## Abstract

Electrospun nanofibers are gaining interest as ocular drug delivery platforms that may adapt to the eye surface and provide sustained release. The aim of this work was to design an innovative ophthalmic insert composed of hyaluronan (HA) nanofibers for the dual delivery of an antioxidant (ferulic acid, FA) and an antimicrobial peptide (ε-polylysine, ε-PL). Polyvinylpyrrolidone (PVP) was added to facilitate the electrospinning process. Fibers with diameters of approx. 100 nm were obtained with PVP 5%-HA 0.8% *w/v* and PVP 10%-HA 0.5% *w/v* mixtures in ethanol:water 4:6 *v/v*. An increase in PVP concentration to 20% *w/v* in both presence and absence of HA rendered fibers of approx. 1 µm. PVP 5%-HA 0.8% *w/v* fibers were loaded with 83.3 ± 14.0 µg FA per mg. After nanofibers crosslinking with ε-PL, blank and FA-loaded inserts showed a mean thickness of 270 ± 21 µm and 273 ± 41 µm, respectively. Blank and FA-loaded inserts completely released ε-PL within 30 min under sink conditions, whereas FA-loaded inserts released the antioxidant within 20 min. Both blank and FA-loaded inserts were challenged against *Pseudomonas aeruginosa* and *Staphylococcus aureus*, demonstrating their efficacy against relevant microbial species.

## 1. Introduction

Electrospinning is becoming a popular technique due to the mild processing conditions and the versatility of substances that can be processed [1]. It may allow manufacturing polymeric fibers with diameter in the nanoscale in a controllable and cost-effective manner with minimum waste of materials [2,3,4]. Electrospun fibers characteristics (diameter, surface properties) can be controlled by tuning process variables (applied voltage, solution flow rate, distance between charged capillary and collector) and polymer variety (MW) and solution properties (composition, viscosity, surface tension, solvent volatility, conductivity, and surface charge density) [1,5]. Electrospun fiber shaped nanostructures have high surface area-to-volume ratio and tunable porosity [2], which may resemble the extracellular matrix. Therefore, this technique has been widely explored for tissue engineering and regenerative medicine applications [6,7,8].

Although less investigated, electrospun nanofibers of natural and synthetic polymers may also find applications as drug delivery platforms, particularly in those cases that thin devices with high specific surface area are required to facilitate the delivery of poorly soluble drugs [9,10]. Drug amorphization in electrospun materials have been proposed to achieve flash or immediate release [11]. Combinations of hydrophilic and hydrophobic polymers and coatings with functional materials have been also tested for endowing the nanofibers with controlled release capabilities [12].

In the ocular field, electrospun nanofibers are gaining interest as soft materials that can easily adapt to cornea and sclera surfaces and remain on the eye surface for a moderate period of time acting as sustained release platforms [13,14,15]. Thus, compared to liquid and semisolid formulations, electrospun inserts may overcome better the precorneal barriers that oppose to ocular bioavailability after topical application [16]. Increased precorneal residence time and higher and prolonged levels of drugs in the aqueous humor have been observed by using nanofibers [17,18,19]. Moreover, nanofibers may create a stable transmembrane drug gradient that facilitates the diffusion through the ocular structures of both hydrophilic and lipophilic drugs [14,20,21,22,23,24,25].

**Figure 1:**
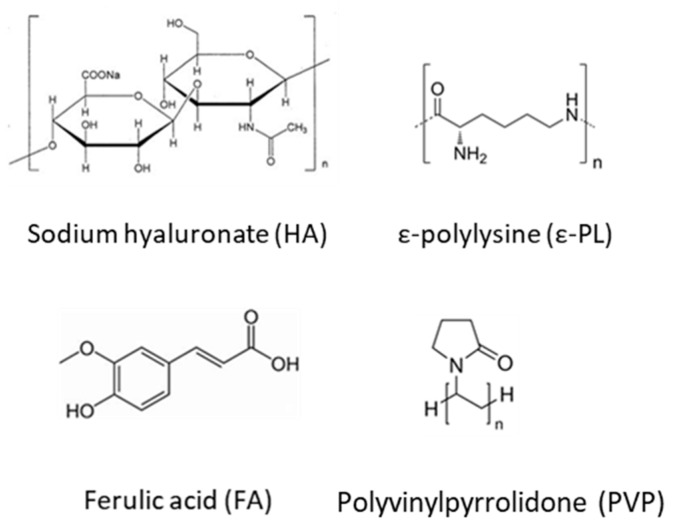
Chemical structures of polyvinyl pyrrolidone (PVP), hyaluronan (HA), ferulic acid (FA) and ε-polylysine (ε-PL).

Hyaluronic acid (HA, Figure 1) is a widely used biopolymer in drug delivery and regenerative field due to its biocompatibility, biodegradability and wound healing properties [26]. This negatively charged glycosaminoglycan, composed of alternating units of D-glucuronic acid and N-acetyl-D-glucosamine, can be hardly electrospun from aqueous solutions due to the high surface tension and chain stiffness that results from the long electrostatic interactions and intramolecular hydrogen bonds, increasing the viscosity. For these reasons, HA has been electrospun in association with other polymers [27,28,29].

Polyvinylpyrrolidone (PVP, Figure 1), another Generally Recognized as Safe (GRAS) excipient, also shows excellent water solubility, biological compatibility, film forming and bioadhesion features [30]. Conversely to HA, PVP nanofibers can be easily electrospun from aqueous solutions [31], although the fibers exhibit rapid disintegration in physiological fluids [32].

Green or natural crosslinking agents have been proposed to prepare HA networks [33]. ε-polylysine (ε-PL, Figure 1) is a water-soluble, biodegradable and GRAS food antimicrobial compound active against bacteria, fungi, and yeast. It is a cationic polyamide naturally produced by the filamentous bacterium *Streptomyces albulus* with a variable number of L-lysine residues bound through amide linkage between ε-amino and α-carboxyl groups [34,35,36]. The antimicrobial activity comprises various mechanisms, such as intercellular reactions, changes in bacterial membrane permeability, interferences with the synthesis of bacterial cell protein and induction of the aggregation of bacterial proteins [36].

The aim of this work was to design ocular inserts of HA-based fibers for ferulic acid (FA, Figure 1) and ε-PL dual delivery to the eye. FA has been recently the subject of intense research interest due to its wide range of therapeutic effects, including anti-oxidant properties, anti-aging, anti-inflammatory, neuroprotective and hematoprotective effects [37,38]. We hypothesized that FA combination with an antimicrobial peptide (ε-PL) could be used for the management of different ocular surface diseases. Due to its physico-chemical properties, HA has been chosen for designing electrospun nanofibers in attempt to modulate FA release to the ocular surface and protect it from light exposure. At first instance, preliminary design and characterization of HA based fibers was performed. Secondly, FA-loaded HA nanofibers were exposed to ε-PL for the simultaneous loading and crosslinking, and the obtained ocular inserts were evaluated in terms of microstructure, release patterns and antimicrobial activity.

## 2. Materials and Methods 

### 2.1. Materials

Epsiliseen®-H (εPL, ε-polylysine, MW 4.7 kDa, Food Grade) was purchased from Siveele B.V. (Breda, Netherlands). Trans-ferulic acid (FA, MW 194.18 Da), Pluronic® F68 (poloxamer 188, PF68, MW 8.4 kDa), polyvinylpyrrolidone (PVP, MW 360 kDa), sodium hyaluronate (HA, MW 600–1100 kDa) and Tryptan blue powder were from Sigma Aldrich (St. Louis, MO, USA). Polyethylene oxide (PEO, MW 200 kDa) was purchased from Thermo Fisher Scientific (Waltham, MA, US). Ethanol absolute AnalaR Normapur® (EtOH, Reagent Ph Eur, Reagent USP) was purchased from VWR Chemicals (Milano, Italia). Water was purified using reverse osmosis (resistivity > 18 MΩ cm, MilliQ, Millipore®, Madrid, Spain). Supragradient HPLC grade acetonitrile and acetic acid glacial (reagent grade) were from Scharlau Chemicals (Barcelona, Spain). NaCl was from Panreac Quimica S.L.U. (Barcelona, Spain).

### 2.2. Nanofibers Preparation and Characterization

#### 2.2.1. Polymer Dispersions

PVP was dissolved in EtOH:water 4:6 (*v/v*) mixture overnight under gentle magnetic stirring at room temperature at different concentrations (2.5%, 5%, 10% and 20% *w/v*). HA was then added to PVP solutions and hydrated under magnetic stirring overnight at room temperature; 20%, 10%, 5% and 2.5% *w/v* of HA were added to 20%, 10%, 5% and 2.5% *w/v* PVP solutions, respectively. The pH of the solutions was close to neutrality.

For FA-loaded nanofibers, the antioxidant was solubilized in a concentration equal to 0.5% *w/v* in EtOH:water 4:6 mixture (*v/v*) by vortexing before addition of PVP (5% *w/v*) and HA (0.8% *w/v*).

#### 2.2.2. Rheology Analysis

Rheological analysis of electrospinning dispersions was performed using a Rheolyst AR-1000N rheometer fitted with an AR2500 data analyzer, a Peltier plate, and a 6-cm in diameter and 2.1° cone geometry (TA Instruments, Newcastle, UK). Viscosity measurements were recorded at 20 °C. Experiments were performed using a single batch for each dispersion.

#### 2.2.3. Electrospinning Process

Solutions (5 mL) were electrospun onto an aluminum grounded target placed at 25 cm from the needle tip. The solutions were placed in a 5 mL plastic syringe of polypropylene connected to a 22-needle gauge (0.7 mm OD × 0.4 mm ID, Aldrich Chemical Co., Saint Louis, MO, USA). The flow rate and high-voltage power source were controlled using a Yflow® Professional Electrospinning Machine (Yflow® S.D., Malaga, Spain). Flow rates were 0.1 mL/h for PVP 2.5%–HA 1% and PVP 5%–HA 0.8% dispersions, 0.5 mL/h for PVP 10%–HA 0.5%, and 0.6 mL/h for PVP 20%–HA 0.2% dispersions. In absence of HA, flow rate of 1 mL/h was used for electrospinning 20% *w/v* PVP solution. Voltages applied to each solution were 15 kV for solutions containing 5%–20% *w/v* PVP, 18 kV for 2.5% *w/v* PVP solution and 25 kV for 10% *w/v* PVP solution (solely or mixed with HA). FA-loaded nanofibers were prepared from FA dispersion (0.5% FA, 5% PVP and 0.8% HA *w/v*) using 0.1 mL/h flow rate and 15 kV voltage. Electrospun fibers were deposited on a piece of aluminum foil above the aluminum collector plate. All experiments were carried out at room temperature and relative humidity of 30%–40%. 

#### 2.2.4. Quantification of FA Loading

Approx. 2 mg of FA-loaded nanofibers were immersed in 1 mL of MilliQ water for 5 min. Then, solutions were 20-fold diluted in MilliQ water and the absorbance recorded at 290 nm (UH5300 UV-Visible Spectrophotometer Hitachi, Chiyoda, Japan) for drug quantification. The experiments were carried out in triplicate.

#### 2.2.5. Nanofibers Morphology

Scanning electron microscope (SEM) images of blank and drug-loaded nanofibers were obtained using a FESEM Ultra Plus (Zeiss, Oberkochen, Germany) at various magnifications. Samples were put onto metal plates and sputter-coated with 10 nm thick iridium film (Q150T-S, Quorum Technologies, Lewes, UK) before viewing.

### 2.3. Cross-Linked Inserts

#### 2.3.1. Nanofibers Crosslinking

Nanofibers-based inserts were prepared by crosslinking with ε-PL. Nanofibers (10 ± 0.5 mg, area ≈ 2 cm^2^, layer thickness ≈ 0.5 mm) were placed on a Petri dish and 0.05 mL ε-PL 50 mg/mL were poured onto the nanofibers. Mixtures were then casted and dried at 37 °C overnight.

#### 2.3.2. Physical Characterization

Inserts thickness was measured using a Caliper Digital Electronic (Fowler™, Newton, MA, USA) and the weight recorded. SEM images of blank and FA-loaded inserts were obtained as described in Section 2.2.5.

#### 2.3.3. FA Release

Blank and FA-loaded inserts (approx. 2.5 mg, 9-mm in diameter disks) were placed in wells containing 2 mL of NaCl 0.9% *w/v* of a 6 well plate at 35 °C under oscillation (100 osc/min, VWR® Incubating Mini Shaker, Spain). Release medium (1 mL) was sampled every 10 min and immediately replaced with the same volume of NaCl 0.9%. FA and ε-PL amounts released were monitored up to 30 min, and samples filtered before drug quantification (0.2 µm hydrophilic PTFE filters, Scharlab S.L., Barcelona, Spain). 

FA was quantified using a reverse-phase Symmetry® C18 cartridge (3.9 × 150 mm, 5 μm, Waters, Milford, MA, USA), thermostated at 30 °C using a HPLC-UV system (Jasco LC Net II/ADC, SpectraLab Scientific Inc., Canada). The mobile phase was a mixture of acetonitrile: 2% acetic acid aqueous solution 19:81 (*v/v*) ratio, pumped at 1 mL/min. The injection volume was 90 μL and the absorbance was recorded at 290 nm. Retention time was 4.7 min. FA calibration curves were built in the range 5–25 µg/mL (R^2^ > 0.999).

ε-PL quantification was performed by using a colorimetric method [39]. Briefly, Tryptan Blue aqueous solution (1 mg/mL, 25 µL) was added to ε-PL standard solutions or release samples (625 µL), and the mixtures incubated at 37 °C for 1 h. Mixtures were then centrifuged at 25 °C for 20 min at 8000 rpm. Finally, supernatants were analyzed at 589 nm (UH5300 UV-Visible Spectrophotometer, Hitachi, Chiyoda, Japan). ε-PL calibration curves were built in the range 0.5–10 µg/mL (R^2^ > 0.992).

### 2.4. Biocompatibility and in vitro Antibacterial Activity Assessment

#### 2.4.1. HET-CAM Test

Hen’s Egg Test Chorioallantoic Membrane (HET-CAM) assay [40] was carried out using fertilized egg (50–60 g; Coren, Spain) previously incubated at 37 °C and 60% relative humidity (climatic chamber) for 9 days. The eggs were turned 3 times per day. The last day they were placed with the wider extreme upward for 12 h and then the eggshell was partially removed (2 cm in diameter) on the air chamber using a rotary saw (Dremel 300, Breda, Netherlands). The inner membrane was wet with 0.9% NaCl for 30 min in the climatic chamber and carefully removed. Pieces of 9-mm inserts were individually placed on the CAM of different eggs. Negative and positive controls were 0.9% NaCl and 0.1 M NaOH solutions, respectively. Photographs of CAM vessels were taken with a digital camera (Canon SX 260HS, without zoom) 5 min after the beginning of the assay and downloaded in the computer in JPEG format. GIMP® software was used to obtain a representative zone of the membrane with the tested formulations.

#### 2.4.2. Fibroblast Compatibility

Fibers and inserts were weighed (4 mg) and added in separate to 1 mL of culture medium. The tubes were vortexed until specimen dissolution and kept at 37 °C. BalB/3T3 cells (CCL-163; ATCC, USA) were cultured at 37 °C in 75 cm^2^ flasks using complete culture medium (DMEM supplemented with 10% *v/v* fetal bovine serum and 1% *v/v* penicillin/streptomycin/fungizone (PSF)) until 80% confluence was reached. Then, cells were trypsinized using TripLE solution (Sigma-Aldrich, MO, USA) and seeded in wells of 96 well plates (5000 cells/well). After 24 h, culture medium was removed and 100 μL of the sample solutions (4 mg/mL) and 100 μL of fresh culture medium were added to each well. Culture medium was used as negative control. Cells were again incubated at 37 °C for 24 or 48 h. At each timepoint, the medium was discarded and the cells were washed with PBS. Immediately after, freshly prepared CCK8 working solution (100 μL) consisting of 90 μL of culture medium and 10 μL of CCK-8 reagent (Sigma-Aldrich, MO, USA) was added to each well and then the cells incubated for 2 h at 37 °C. Finally, absorbance (450 nm) was recorded using a microplate reader (Model 8; BioRad, CA, USA).

#### 2.4.3. Antibacterial Activity

The antibacterial activity was evaluated on Müller–Hinton agar plates seeded with *Pseudomonas aeruginosa* (CEC T110; 3.4∙10^9^ CFU/mL) or *Streptococcus aureus* (ATCC 25923; 1.2∙10^9^ CFU/mL). Blank and FA-loaded inserts were placed on the inoculated agar plates and the zones of inhibition were measured after incubation at 35 °C for 24 h. All experiments were carried out in duplicate.

### 2.5. Statistical Analysis

All data are reported as mean value ± sd. Differences were analyzed using ANOVA and multiple range test (Statgraphics Centurion XVI 1.15, StatPoint Technologies Inc., Warrenton, VA, USA). Differences were considered statistically significant when *p* < 0.05.

## 3. Results and Discussion

The present study was aimed at designing an ophthalmic platform able to increase FA residence time on the ocular surface, assuring in the meantime ε-PL release to the eye. Ophthalmic inserts could offer an increased ocular residence, controlled drug release and accurate dosing. The improved residence time of the drug in the conjunctival sac may enhance drug ocular availability with less frequent administration and fewer side effects than eyedrops [41].

### 3.1. HA Based Nanofibers

HA based nanofibrous membranes have been previously investigated as biomimetic tissue engineering scaffolds, wound healing materials, and drug delivery systems [42]. However, electrospinning of pure HA for scaffold preparation has been largely unsuccessful due to its inadequate physicochemical properties [29]. Indeed, high viscosity and surface tension hinder the electrospinning process and favor the fusion of electrospun nanofibers due to slow evaporation of the solvent [42]. It has been also reported that chain conformation of HA should change from the rigid alpha helix structure to the coil conformation for a successful electrospinning process [43]. Commonly, electrospinning requires polymers of quite high molecular weight and at a concentration above the critical overlapping value to facilitate chain entanglement in the dispersion and thus fiber formation during the electrical field-reduced flow [44]. Nevertheless, if polymer solution concentration is very high, bead fibers with thick diameters are formed because of the short drying time [45]. 

High MW sodium hyaluronan (MW 600–1100 kDa) was chosen for preparing electrospinning solutions at low concentrations. Preliminarily, electrospinning of HA aqueous dispersion was investigated. In accordance to literature [42], fibers solely were not obtained because of the instability of the jet (liquid atomization process, namely electrospraying). Thus, a small percentage of EtOH was added during the preparation of HA solutions to reduce the time needed for the solvent evaporation during electrospinning process. However, the addition of EtOH up to 20% *v/v* during the preparation of HA solutions (0.1%, 0.05% and 0.02% *w/v*) did not ameliorate the electrospinning process and the Taylor cone was not stable.

Incorporation of other chemical compounds was also investigated to overcome unfavorable physicochemical properties of HA solutions. Ionic surfactants may increase the net charge density and the instability of the charged jet leading to finer fibers [10]. The addition of 5% *w/v* Pluronic F68 to 0.2% *w/v* HA in 20% EtOH: 80% water and 40% EtOH: 60% water mixtures (*v/v*) did not lead to stable Taylor cone either. Similar results were observed by using 0.5% *w/v* HA concentration under the same conditions. The addition of PEO 200 (12% *w/v*) to 0.2%, 0.3%, 0.5%, 0.8% *w/v* HA aqueous solution did not improve the process either.

Next attempt was carried out with high molecular weight PVP (360 kDa) due to its already demonstrated capability to render electrospun nanofibers [33]. HA:PVP ratio and electrospinning parameters were adjusted to obtain a stable Taylor cone and consequently bead-free fibers with a sub-micron diameter. Particularly, various polymers concentrations were tested as fibers diameters could be highly dependent on this parameter and dispersion viscosity [5].

**Figure 2:**
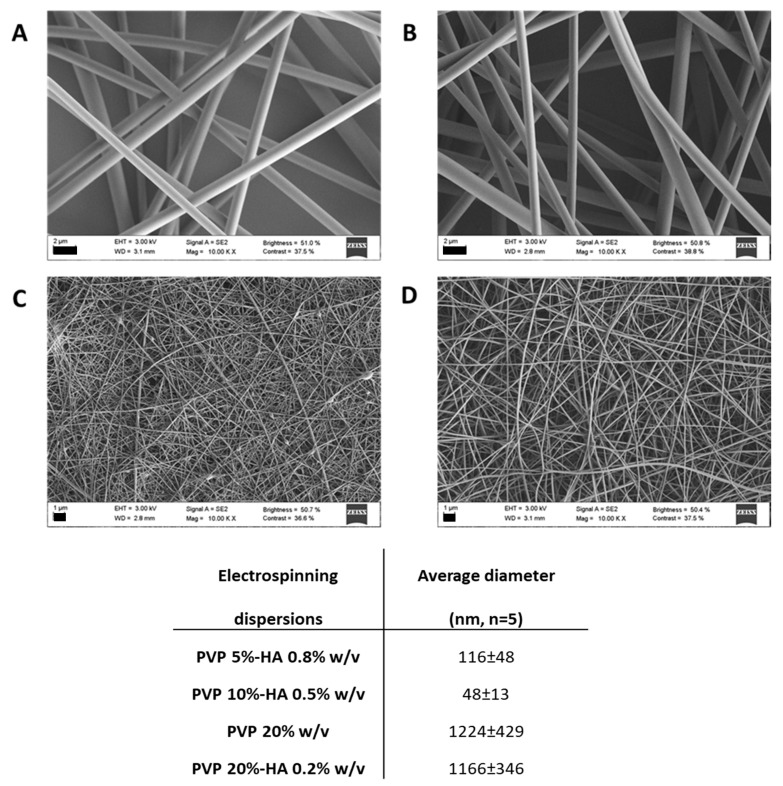
SEM micrographs of PVP-HA nanofibers electrospun at 10 kV (A; PVP 20%, B; PVP 20%–HA 0.2%, C; PVP 10%–HA 0.5%, D; PVP 5%–HA 0.8%, scale bars 2 µm for A-B and 1 µm for C-D).

SEM micrographs of electrospun fibers prepared varying PVP and HA concentrations in EtOH:water 4:6 *v/v* are shown in Figure 2. Particles were detected in the case of PVP 2.5%–HA 1% *w/v* dispersions (not shown). Fibers with diameters of approx. 100 nm were obtained for 5% and 10% *w/v* PVP concentration; namely PVP 5%–HA 0.8% *w/v* and PVP 10%–HA 0.5% *w/v* had average diameters of 116 ± 48 and 48 ± 13 nm. An increase in PVP concentration to 20% *w/v* in both presence and absence of HA rendered fibers of approx. 1 µm; namely, the average diameters of PVP 20% *w/v* and PVP 20%–HA 0.2% *w/v* were 1224 ± 429 and 1166 ± 346 nm. The fibers showed homogenous surface indicating that all the process parameters were well controlled.

**Figure 3:**
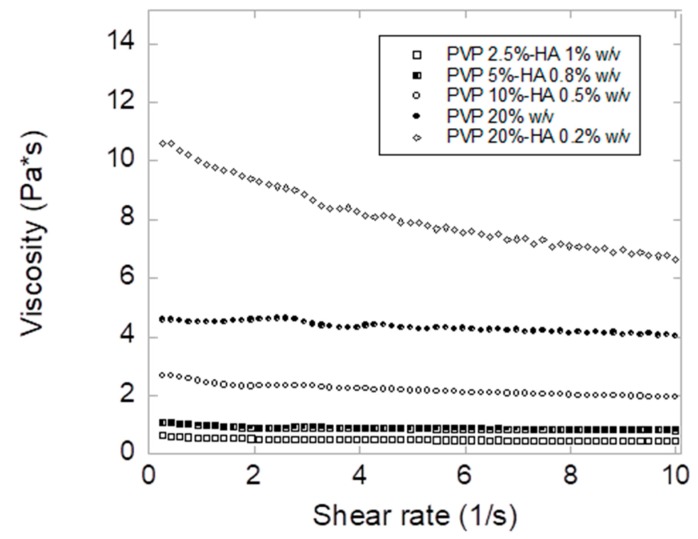
Viscosity measurements of electrospinning dispersions recorded at 20 °C.

The high increase in fiber average diameter observed for PVP 20% *w/v* and PVP 20%–HA 0.2% *w/v* (Figure 2) is explained by the increase in viscosity (Figure 3) [32]. For drug loading, PVP 5%–HA 0.8% *w/v* dispersion was selected due to the sub-micron diameters of the obtained fibers and beads absence.

**Figure 4:**
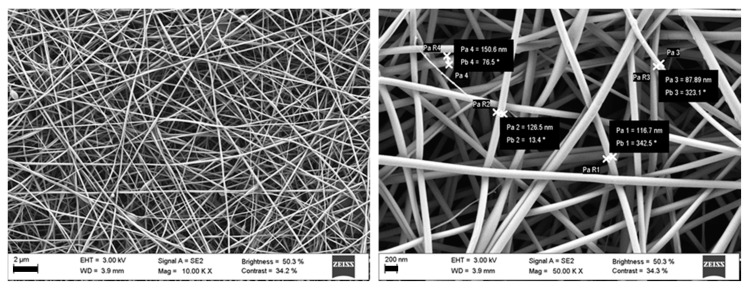
SEM of FA-loaded nanofibers obtained by electrospinning 0.5% FA, 5% PVP and 0.8% HA *w/v* dispersion (scale bars 2 µm on the left and 200 nm on the right).

SEM of blank and FA-loaded nanofibers were comparable in morphology, demonstrating that FA incorporation did not have an impact on fiber morphology (Figure 4) and diameter (120 ± 26 nm). Additionally, the content in FA of the nanofibers was checked after electrospinning process (83.3 ± 14.0 µg/mg of fiber vs. theoretical 80 µg/mg of fiber) and confirmed that the application of high voltage field did not cause FA degradation.

### 3.2. Ocular Inserts of Nanofibers Crosslinked with ε-polylysine

Further studies were carried out with PVP 5%–HA 0.8% *w/v* nanofibers which were crosslinked with ε-PL taking benefit of the electrostatic interactions among the two polymers. Indeed, ε-PL is able to interact by electrostatic interactions with anionic polymers, and HA and ε-PL interactions have been already exploited for obtaining polyelectrolyte multilayers [46,47,48]. Apart from layer-by-layer structures, ε-PL is expected to interact with hyaluronan thanks to inter- and intra- molecular linkages between ε-PL amino groups and hyaluronan carboxylic groups forming macro-, micro- or nanohydrogels. In this study, electrostatic interactions were used to favor the crosslinking bonds between HA nanofibers to obtain inserts to be placed in the conjunctival sac.

Different mass ratios were tested by varying the concentration and the volume of the ε-PL solution to be added to HA nanofibers. As both PVP and HA are highly hydrophilic polymers, high concentration and small volume of the ε-PL solution to be added was selected (Fiber/ε-PL mass ratio equal to 2.4).

**Figure 5:**
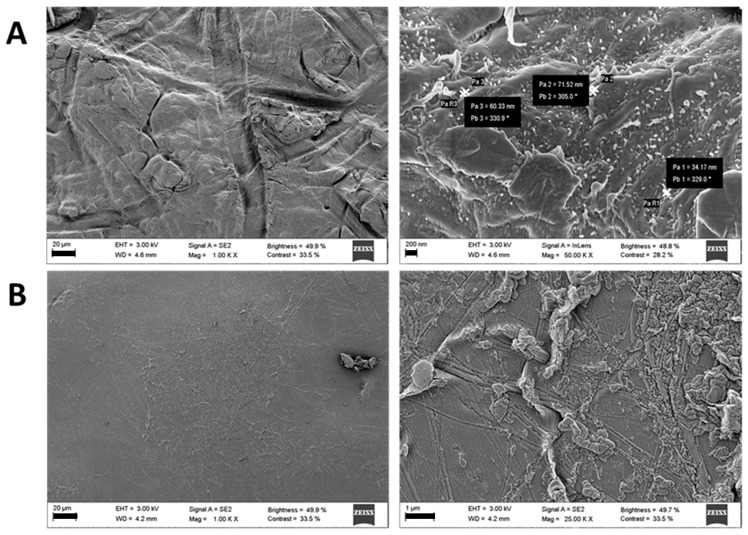
SEM micrographs of blank (**A**) and ferulic acid loaded (**B**) inserts (scale bars 20 µm on the left, 200 nm on the right at the top and 1 µm on the right at the bottom).

After crosslinking blank nanofibers, inserts appeared white with a mean thickness of 270 ± 21 µm and a weight of 39.5 ± 11.2 µg/cm^2^. Conversely, crosslinked FA-loaded inserts appeared light yellow showing a mean thickness of 273 ± 41 µm and a weight of 46.8 ± 8.8 µg/cm^2^. SEM images of blank cross-linked inserts revealed the presence of nanofibers structures even after electrostatic crosslinking, whereas these structures seemed not to be conserved on inserts surface in case of FA- loaded inserts (Figure 5) presumably due to electrostatic interactions between FA and the cross-linker ε-PL.

**Figure 6:**
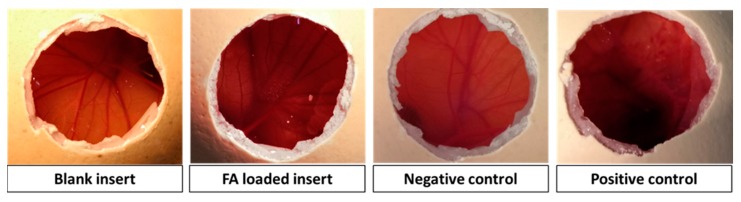
HET-CAM photographs after 5 min from the beginning of the test with blank and ferulic loaded inserts, and negative (0.9% NaCl solution) and positive (0.1 N NaOH solution) controls.

First screening of the ocular compatibility of the inserts with ocular tissues was assessed by HET-CAM assays. The chorioallantoic membrane of an embryonated hen’s egg resembles the vascular conjunctiva of the eye. Thus, irritating effects after conjunctiva exposure of inserts can be predicted from changes in the chorioallantoic membrane [49]. In our case, none insert caused haemorrhage, vessels lysis or coagulation, and performed the same as the saline solution control (Figure 6). Adverse events occurred only in case of the positive control (NaOH 5 M), resulting in a rosette-like coagulation. Overall, HET-CAM results indicated that the designed inserts can be considered as non-irritant. These results were confirmed with a cytocompatibility study carried out with fibroblasts, which revealed cell viability values above 80% after 24 and 48 h in contact with blank and FA-loaded fibers and inserts.

**Figure 7:**
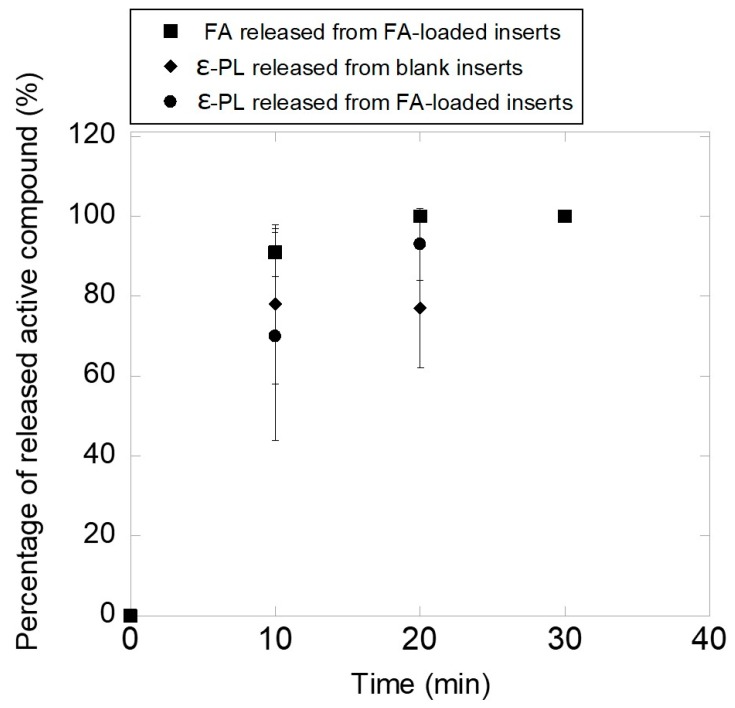
FA and ε-PL release patterns from blank and FA loaded inserts (mean ± sd, n = 3).

Then, the inserts were tested regarding their capability to release both FA and ε-PL. Although no specific methods are reported in Pharmacopoeias for studying drug release from ophthalmic dosage forms, in vitro studies under sink conditions in NaCl 0.9% medium may be useful as first step for studying the behavior of ophthalmic drug delivery systems [50]. The weight of the inserts was adjusted to have the same amount of FA and ε-PL. Blank and FA-loaded inserts eroded upon contact with saline solution in a short time interval (under the oscillation conditions tested) without noticeable swelling, and thus FA was released completely in 20 min. As shown in Figure 7, FA-loaded inserts (loading equal to 5.7% ± 0.2% *w/w*) released 91% ± 6% in 10 min and 100% of the loaded drug in 20 min. Concerning ε-PL loading, FA presence did not affect the peptide concentration into the inserts (12.2% ± 2.8% and 17.6% ± 3.1% w/w for blank and FA-loaded inserts) neither the peptide release. Blank inserts released 78% ± 20%, 77% ± 15% and 100% of ε-PL in 10, 20 and 30 min, respectively. Similarly, FA-loaded inserts released 70% ± 26%, 93% ± 9% and 100% of peptide in 10, 20 and 30 min, respectively. It should be noticed that under physiological conditions the volume of tears is limited and thus the insert could hydrate, adhere to the mucus on the ocular surface and release the drug more slowly [51,52]. Strong antioxidant activity of FA has been reported for concentrations of 1 mg/mL [53], which could be provided by the inserts (0.14 mg FA in the 2.5 mg insert) on the ocular surface in few minutes after application. 

**Figure 8:**
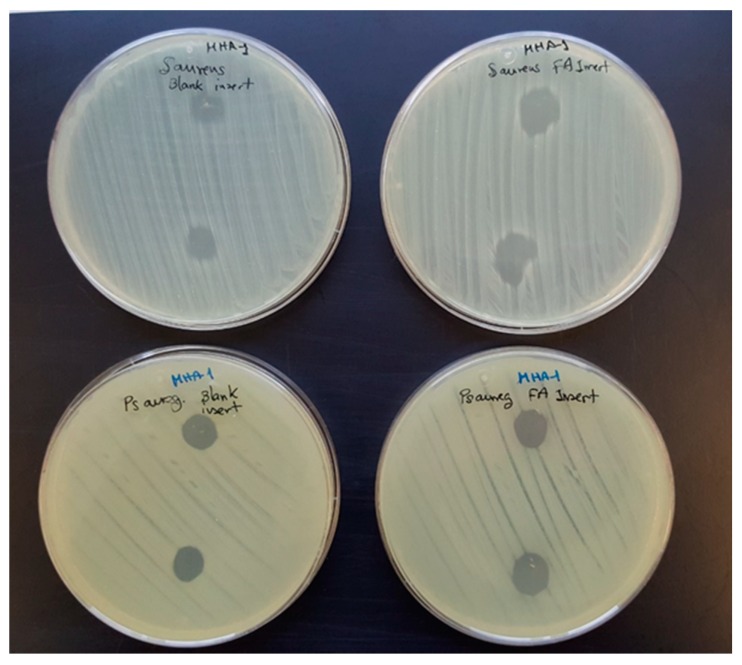
*Staphylococcus aureus* (first row) and *Pseudomonas aeruginosa* (second row) inhibition zones caused by blank and FA-loaded inserts.

Lastly, blank and FA-loaded inserts were challenged against *Pseudomonas aeruginosa* and *Staphylococcus aureus* and the growth inhibition zones (Figure 8) were measured after 24 h of incubation. Differently to the starting fibers which did not show any antimicrobial activity (data not shown), blank and FA-loaded inserts resulted effective against both microbial species. This antibacterial efficacy can be ascribed to the presence of the peptide into the inserts. Indeed, the antibacterial mechanism of ε-PL against Gram-positive and Gram-negative bacteria has been attributed to membrane disruption related to the interactions of the primary amine surface groups with the cell membrane [54,55]. However, a solution prepared with the PL concentration expected to be provided by the inserts did not cause growth inhibition. Although the reasons for this finding are not clear, they may be related to the fact that the insert retains PL on its surface while the solution may freely diffuse to the agar medium and become diluted and thus inefficient. 

## 4. Conclusions

HA nanofibers were successfully obtained in presence of PVP, showing proper dimensions and ferulic acid loading. Crosslinked inserts were obtained by crosslinking nanofibers with ε-PL and evaluated for a possible ocular application. Crosslinked inserts showed adequate thickness, release pattern, in vitro biocompatibility and antibacterial activity. Further ex vivo and in vivo studies (in particular, the permanence time of the formulation on sclera/conjunctiva and FA permeability) may contribute to forecast the suitability of the crosslinked inserts for ocular application.
